# Right atrial approach for repairing a posterior ventricular septal rupture: a case report

**DOI:** 10.1186/s40792-016-0215-9

**Published:** 2016-08-30

**Authors:** Katsukiyo Kitabayashi, Keisuke Miyake, Nobuo Sakagoshi

**Affiliations:** Department of Cardiovascular Surgery, Kinan Hospital, Shinjo-cho 46-70, Tanabe, 646-8588 Wakayama Japan

**Keywords:** Mechanical complication, Acute myocardial infarction, Posterior ventricular septal rupture, Right atrial approach

## Abstract

**Background:**

Ventricular septal rupture (VSR) is a life-threatening complication following acute transmural myocardial infarction. Posteriorly located ruptures are one of the main predictors of poor prognoses because of the surgical difficulties associated with this location.

**Case presentation:**

A 72-year-old man with a posterior VSR underwent surgical repair via the right atrial approach. The patient’s postoperative course was uneventful, and echocardiography showed no residual shunt flow. He was discharged on postoperative day 37.

**Conclusion:**

By temporally detaching the tricuspid valve leaflet, this approach provides a better view and handling space within the posterior ventricular septum than the trans-ventricular approach. Additionally, avoiding a ventricular incision can better preserve postoperative ventricular function.

## Background

Ventricular septal rupture (VSR) is a life-threatening complication following transmural myocardial infarction [1]. Although several surgical procedures for VSR have been reported and the outcomes of these challenging procedures have been improved, the mortality and morbidity rates are still high.

Compared to anterior VSRs, posterior VSRs are a major predictor of poor prognoses [2] because of the technical difficulties associated with approaching the deep basal ventricular septum.

However, the right atrial approach can overcome this problem, providing a better view of the posterior ventricular septum [3, 4].

Here, we report the case of a patient with a post-infarction posterior VSR that was repaired via the right atrial approach.

## Case presentation

**Fig. 1:**
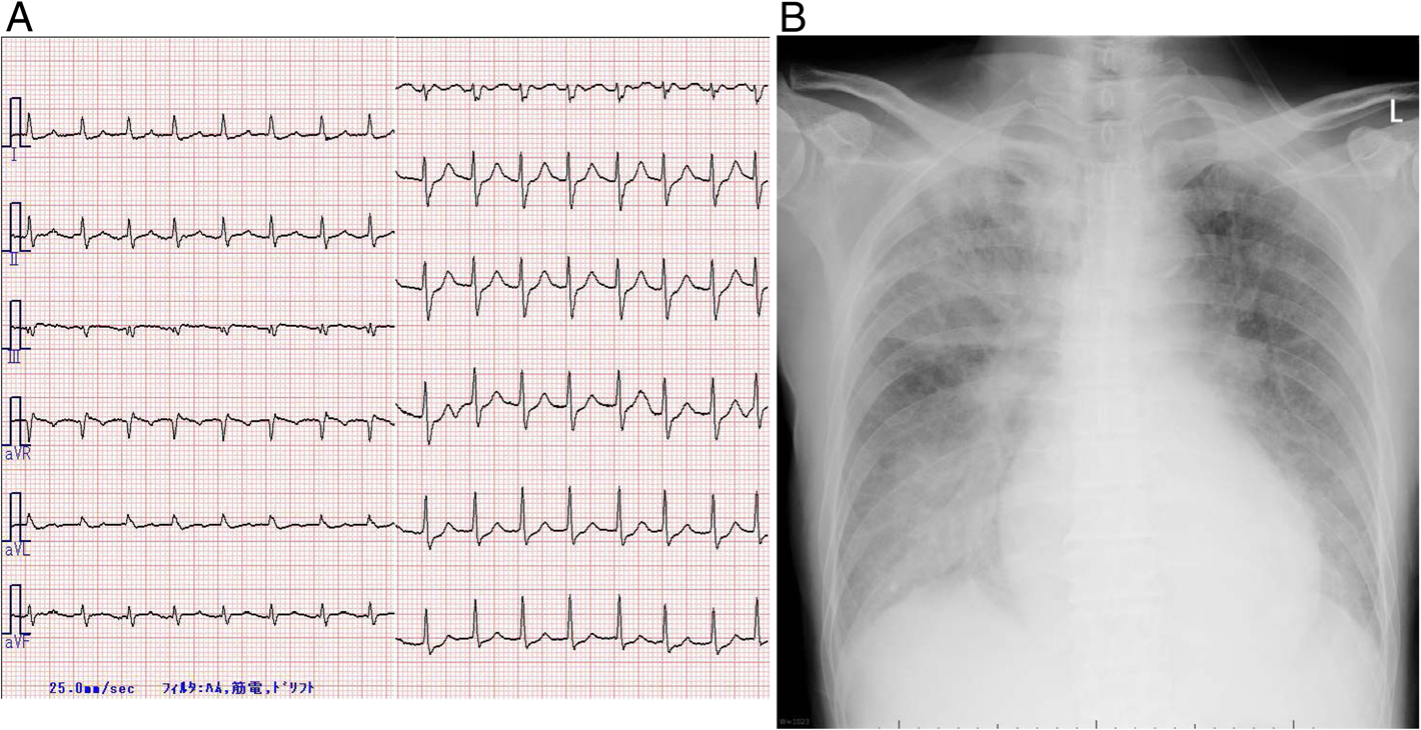
Preoperative examinations. **a** Electrocardiogram: atrial tachycardia (150 beats/min) with no apparent ST elevation. **b** Chest roentgenogram: severe congestion of the lung and moderate pleural effusion

A 72-year-old man was referred to our hospital with dyspnea on effort and palpitations. Although he had no history of chest pain, he felt nauseous and had experienced dizziness for 6 days before hospitalization. An electrocardiogram showed atrial tachycardia (150 beats/min) with no apparent ST elevation (Fig. 1a). Echocardiography revealed no left ventricular asynergy, and the patient’s serum creatine kinase level was within the normal range (174 IU/l). We therefore did not suspect acute coronary syndrome at admission.

**Fig. 2:**
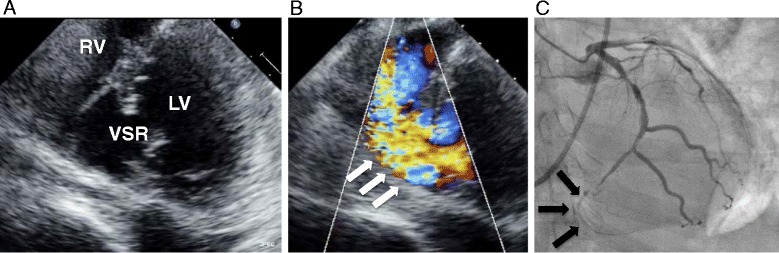
Preoperative examinations. **a** Short-axis view of preoperative transthoracic echocardiography showing a perforation site (17 mm in diameter) near the posteromedial papillary muscle in the basal ventricular septum. **b** Left-to-right shunt flow via the ventricular septal rupture in the systolic phase (*white arrow*). **c**. Coronary angiography showing complete occlusion of the last branch of the dominant left circumflex artery (*black arrow*)

However the patient’s circulatory and respiratory status deteriorated even with optimal medication (inotropes and a vasodilator). A chest roentgenogram showed severe congestion of the lung and a moderate amount of effusion in both pleural cavities (Fig. 1b). Enhanced computed tomography suggested shunt flow in the cardiac cavity. A second echocardiogram revealed a VSR in the basal part of the posterior ventricular septum with left-to-right shunt flow (Fig. 2a, b). Emergency coronary angiography revealed occlusion of the final small branch of the dominant left circumflex artery (Fig. 2c).

During angiography, an intra-aortic balloon pump (IABP) was used to support coronary flow and cardiac function. Postinfarction posterior VSR was diagnosed, and emergency surgery was performed.

**Fig. 3:**
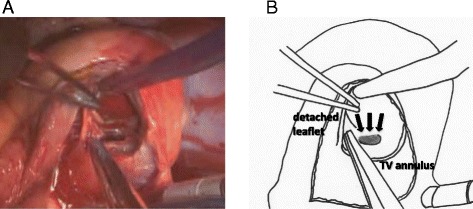
Operative findings. **a** Surgical view of the right atrial approach after the tricuspid valve leaflet was detached. **b** Diagram of **a**. Ventricular septal rupture = *black arrow*

After median sternotomy, cardiopulmonary bypass was established in the usual manner. After aortic cross clamping and cardiac arrest, we made a right atrial oblique incision. We located the perforation site in the basal posterior part of the ventricular septum (size, 17 mm) (Fig. 3a, b). It was just behind the septal leaflet of the tricuspid valve; thus, a part of the septal and posterior leaflet of the tricuspid valve was detached from the annulus to obtain a better view and handling space.

After resecting the necrotic tissue around the defect, the edge of the defect was not friable, which suggested that the infarction was not large. We closed the VSR using the double patch technique. The left and right sides of the ventricular septum were covered with a bovine pericardial patch with eight interrupted mattress sutures (4-0 polypropylene). The tricuspid valve leaflet was then re-sutured to its annulus using a continuous suture (5-0 polypropylene), and the right atrium was closed.

After declamping the ascending aorta, we successfully weaned the cardiopulmonary bypass using IABP support. The patient was moved to the intensive care unit on an IABP and mechanical ventilation.

On the first postoperative day since the patient’s cardiac and respiratory functions were stable, we discontinued IABP support and performed extubation. Postoperative echocardiography showed no residual shunt flow, almost normal ventricular wall motion, and a trivial degree of tricuspid valve regurgitation.

After his atrial arrhythmia and junctional bradycardia were controlled, the patient was discharged on postoperative day 37.

## Conclusions

VSR is a life-threatening complication that occurs in 1–2 % of patients following acute transmural myocardial infarction [1]. Surgical intervention is necessary because of the poor natural history of the entity, and several operative procedures have been reported in the literature.

Daggets et al. reported infarct resection and suturing of the right and left ventricles, including the ventricular septum [5]. After 1990, the infarct exclusion technique with an incision of the left ventricular anterior free wall became popular and resulted in improved surgical results [6]. The double patch technique via the right ventricle was reported to be an alternative approach for avoiding a left ventricular incision and providing a better surgical view with the help of intra-operative epicardial echo [7].

Although the outcomes of VSR repair have improved in recent years due to advancements in myocardial protection during surgery and intensive postoperative care, the mortality rate is still high (20–35 % for 30-day mortality and 55–63 % survival at 5 years) [2]. The reported risk factors affecting the prognoses of VSRs include preoperative factors such as preoperative condition and posterior VSR and postoperative factors such as residual shunt flow and ventricular arrhythmia. Posterior VSRs, in particular, are technically difficult to repair because of their location. Suturing within the deep basal septum is complicated by the use of an apical ventricular incision.

VSR repair using a right atrial approach has been reported to overcome these difficulties [3, 4]. This approach provides a better view of the basal septal defect in case of posterior myocardial infarction. This approach is commonly used for congenital ventricular septal defects, but surgeons rarely use it for acquired VSRs.

The reason for this is that in VSR repair, the sutures must be placed in the fragile edge of the infarcted ventricular septum, and a large patch is sometimes needed to cover the defect. It is complicated to suture into the deep apex of the ventricular septum from the right atrium.

However, separating the tricuspid valve leaflet widely, not only the septal leaflet but also part of the posterior leaflet, improves the surgical view of the ventricular septum. We believe that concomitant tricuspid valve replacement will expand the indication of this technique.

In more serious cases, in which the infarct is too large and suturing of the deep apex area is necessary, an additional ventricular incision to the right atrial incision can be incorporated during surgery.

Posterior VSRs mainly caused by single-branch infarctions of the left circumflex artery or right coronary artery. When the middle to apex part of the ventricular septum is preserved with perfusion from the left anterior descending branch on preoperative echocardiography, the right atrial approach is suitable for performing repairs.

In the present case, the infarct was relatively small and the last branch of the left circumflex artery was occluded, making the right atrial approach mostly suitable for approaching the whole edge of the VSR.

In addition, the right atrial approach can improve many of the postoperative factors associated with poor prognoses. Remodeling of the infarcted myocardium and making a large incision in the ventricular free wall affect postoperative low ventricular function and ventricular arrhythmia. A large suture line and tight closure of the ventricle also reduce the residual ventricular volume and worsen ventricular diastolic function [8].

The right atrial approach eliminates the need for a ventricular incision and re-suturing, thus allowing the surviving myocardium and cardiac function to be preserved. In addition, the new onset of ventricular arrhythmia can be avoided.

Performing trans-catheter VSR repair with an occluding device is now possible. Currently, this less invasive technique is only recommended for small VSRs (<1.5 mm) and in subacute or chronic settings [9]. Larger defects and friable surrounding myocardium increase the risk of the device migrating and some of the VSR being left behind, which can result in unfavorable prognoses.

Although surgeons must be cautious of possible postoperative tricuspid valve regurgitation and atrial arrhythmia, the right atrial approach is a useful method for posterior VSR repair.

In conclusion, we performed posterior postinfarction VSR repair via the right atrial approach. This approach is feasible for repairing posterior VSRs because it provides better exposure of the posterior septal defect than the trans-ventricular approach. Moreover, it allows postoperative ventricular function and ventricular arrhythmia to be avoided.

## Abbreviations

IABP: Intra-aortic balloon pumping
VSR: Ventricular septal rupture

## References

[1] Kouchoukos N, Blackstone E, Doty D, Hanley F, Karp R (2003). Kirklin/Barratt-Boyes cardiac surgery.

[2] Fukushima S, Tesar PJ, Jalali H (2010). Determinants of in-hospital and long-term surgical outcomes after repair of postinfarction ventricular septal rupture. J Thorac Cardiovasc Surg.

[3] Filgueira JL, Battistessa SA, Estable H (1986). Delayed repair of an acquired posterior septal defect through a right atrial approach. Ann Thorac Surg.

[4] Massetti M, Babatasi G, Le Page O (2000). Postinfarction ventricular septal rupture: early repair through the right atrial approach. J Thorac Cardiovasc Surg.

[5] Daggett WM, Burwell LR, Lawson DW (1970). Resection of acute ventricular aneurysm and ruptured interventricular septum resection of acute ventricular aneurysm and ruptured interventricular septum after myocardial infarction. N Engl J Med.

[6] Komeda M, Fremes SE, David TE (1990). Surgical repair of postinfarction ventricular septal defect. Circulation.

[7] Isoda S, Imoto K, Uchida K (2004). Sandwich technique via right ventricle incision to repair postinfarction ventricular septal defect. J Card Surg.

[8] Castelvecchio S, Menicanti L, Ranucci M (2008). Impact of surgical ventricular restoration on diastolic function: implications of shape and residual ventricular size. Ann Thorac Surg.

[9] Attia R, Blauth C (2010). Which patients might be suitable for a septal occluder device closure of postinfarction ventricular septal rupture rather than immediate surgery?. Interact Cardiovasc Thorac Surg.

